# Investigating the effect of abutment–implant connection type on abutment screw loosening in a dental implant system using finite element methods

**DOI:** 10.15171/joddd.2019.044

**Published:** 2019

**Authors:** Alireza Pournasrollah, Ramin Negahdari, Vahedeh Gharekhani, Ali Torab, Soheil Jannati Ataei

**Affiliations:** ^1^Department of Prosthodontics, Faculty of Dentistry, Tabriz University of Medical Sciences, Tabriz, Iran; ^2^Department of Prosthodontics, Faculty of Dentistry, Urmia University of Medical Sciences, Urmia, Iran; ^3^Department of Prosthodontics, Faculty of Dentistry, Golestan University of Medical Sciences, Golestan, Iran

**Keywords:** Finite element method, implant–abutment connection, micro-motion, stress distribution

## Abstract

***Background.*** The most common problem associated with dental implants is the abutment screw loosening. This research aimed to investigate the effect of the type of connection on screw loosening, using a finite element method (FEM).

***Methods.*** Periosave system and different types of the implant–abutment connection were used for modeling. After being measured, CAD files were modeled using CATIA software and imported to the ANSYS analysis software, and the model was loaded.

***Results.*** A force of 100 N was applied at 0.1 second, and no force was applied at 0.42 second. The screw head deformation at 0.1 and 0.42 seconds was 8 and 3.8 μm, and 7.6 and 2.8 μm at morse taper and octagon dental implant connections, respectively. The displacement rate of the internal surface of the abutment at 0.1 and 0.42 seconds was 10.7 and 8.4 μm, and 5.7 and 5.6 µm in the octagon and morse taper dental implant connections, respectively. The displacement of the implant suprastructure–abutment interface from the screw head at 0.1 and 0.42 seconds was 9 and 7 μm, and 7 and 6 μm in the morse taper and octagon dental implant connections, respectively. At intervals of 0 to 0.1 seconds and 0.6 to 0.8 seconds, the octagon connection was separated at the maximum screw head displacement and the internal part of the abutment, but the morse taper connection did not exhibit any separation. In the above time intervals, the results were similar to the maximum state in case of the minimum displacement of the screw head and the internal part of the abutment.

***Conclusion.*** Screw loosening is less likely to occur in the morse hex connection compared to the octagon connection due to the lack of separation of the screw from the internal surface of the abutment.

## Introduction

Titanium endosteal implants, which are osseointegrated, are widely used for their mechanical advantages and excellent bone connection. Long-term follow-up studies have shown many complications after the prosthetic phase of treatment, such as the loss of osseointegration, abutment screw loosening, abutment screw fracture, and other problems.^1^ The most common problem is abutment screw loosening and fracture, especially in single-tooth implants. A loose screw can lead to crestal bone resorption.^1-3^ Screw loosening is costly and time-consuming. Screw copings are usually the weakest connection in the prosthetic chain. Any kind of occlusal, casting, or force inconsistency can cause screw loosening or fracture.^2,4^ When the screw is tightened for the first time, an initial tensile preload is created within the screw. Preloading leads to the imposition of pressure on the abutment–implant components and friction between the screw and the threads of the implant, screw head, and abutment, as well as the upper part of the implant and the lower part of the abutment. This pressure causes resistance to the external shearing forces and increases the fatigue strength of the connection.^2-4^ Preload is created in the screw by creating cracks.^4^ The settling effect (embedment relaxation) is another effective factor. When the abutment is placed in the implant, the settling effect occurs by applying torques, which increases due to the micro-roughness between the metal surfaces of the implant and the abutment. Abrasion of the contact surfaces brings the two surfaces closer.^5^ The settling effect eliminates the initial preload by 2–10%.^5^ In the past, implants with Branemark's external hexagon connections were used, which resulted in complications, such as screw loosening and rotational misfit, and the resultant microbial penetration led to changes in the external hexagon and internal connection.^2^ External and internal connections are distinguished based on the presence or absence of the appendage extending out of the implant body. In external connections, an appendage is found that extends out of the implant body, while this appendage is placed in the internal side of the implant body in implants with internal connections.^3^ There is an anti-rotational feature for the abutment connection in internal connections. The most common designs include hexagonal, octagonal, morse taper, and hex morse taper.^1,3,4^ The internal hexagonal implants are the most commonly available type, with a hexagonal shape. The morse taper implants include a tapered design in the abutment design that is inserted into the indentation of the tapered implant. This type of connection depends on the friction fit to remove the rotation at the abutment–implant interface and prevent screw loosening. This type of connection also causes resistance against bending forces. Internal octagonal implants present as octagonal connections.^6,7^ Implant–abutment connections or prosthetic connections with unstable coupling surface exert unwanted stresses on the fastening screw.^2^ Khraisat et al^8^ investigated the effect of lateral cyclic loading on the abutment screw loosening in the external hexagon implants. Simon^9^ investigated the success rate of implant restorations of the single implant-supported molar and premolar crowns, and the results showed that the implant failure rate was 4.6%, of which 7% was related to the abutment screw loosening. Binon^10^ observed a direct relationship between the rotational misfit of hexagon implants and screw loosening. Since conducting clinical studies on screw loosening is a time-consuming and costly process, studies should be carried out using a systematic approach. This approach is the finite element method (FEM), a powerful and effective method to predict the mechanical behavior of implant restorations.^1^ Although most of the FEM-based implant-supported dental prosthesis analyses have been performed under static conditions, the dynamic response of dental implants has been studied less frequently. Since the dental loading model is transient in nature, the nonlinear and transient dynamic analysis is widely used in industrial fields; this analysis is also expected to predict the dynamic behavior of dental implants. There are few reports on the FEM components for the evaluation of dental implants.^1^

### Transient dynamic analysis

With the help of this analysis (sometimes referred to as transient time history [TTH]), one can calculate the dynamic responses of one structure under the influence of time-dependent loading. In this analysis, we can calculate displacements, strains, and stresses and time-dependent forces in a structure. The main equation of a transient dynamic analysis is as follows:

MẌ+CẊ+KX=F(t)

where M is the matrix of the system mass, C is the damping matrix, K is the stiffness matrix, Ẍ is the acceleration vector, Ẋ is the velocity vector, X is the displacement vector, and F_(t)_ is the time-dependent load vector.

## Methods

**Figure 1:**
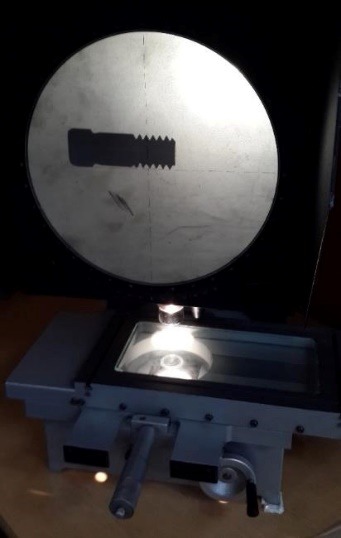
The profile projector.

**Figure 2:**
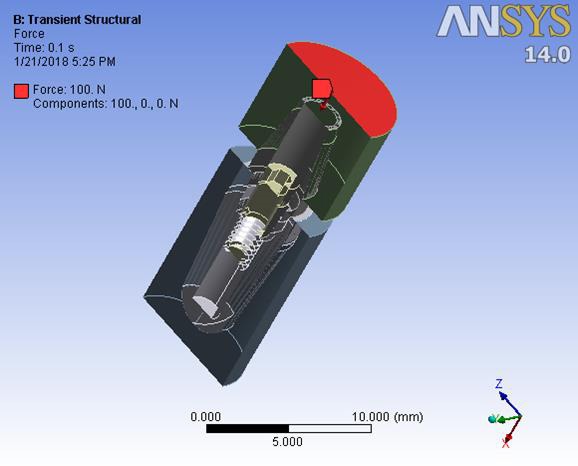
The steps of defining the dynamic axial force on suprastructure 1.

**Figure 3:**
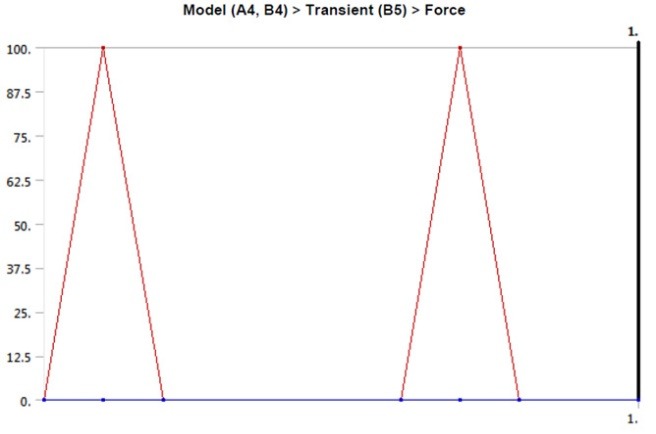
The suprastructure of the dynamic loading model.

The Periosave system was used for modeling of the implant and abutment fixture, and the implant and abutment with the usual diameters of 4 mm and 4.5 mm were selected. In order to imitate the clinical conditions, the height of the abutments was adjusted to 5 mm. Implants, abutments, abutment screws, and connections were accurately measured using a profile projector, and their real dimensions were later obtained (Figure 1). The device resolution is about 0.01 mm, and the ×50 magnification was used. The practical construction of resin suprastructures was carried out by autopolymerizing resin (Pattern Resin; GC Corporation, Tokyo, Japan). The resin fully covered the surface of the abutments, and the thickness of the resin layer was approximately 2 mm. Then, the resin was cylindered inside a phosphate-bonded casting investment (Ticonium, Albany, NY, USA), and casting was later carried out by a nickel–chromium alloy (BEGO, Germany). Subsequently, a suprastructure scan was performed by the profile projector instrument. All the analyzed components were transferred to ANSYS software separately in an assembled manner, and the connection method was defined using contact elements. In this section, we defined the type of connection between the components as well as the friction coefficients between them by taking into account the nature of the connection. Since four pieces, i.e., implants, screws, abutments, and suprastructure, are used in the montage structure analysis, it is necessary to define the real relationship and connection between these components in the ANSYS software and carry out the analysis accordingly. The implant–screw, implant–abutment, and screw–abutment connections are of friction type, and abutment–suprastructure connection is considered as a complete connection without displacement (Table 1).  The intended preloading force was 400 N, equivalent to 30 N.cm torque, according to the reference information. Taking into account the boundary conditions, all the environmental nodes surrounding the bone were free of motion and fixed to prevent the movement of the model during the force application process. The mechanical properties of each component were introduced to the system (Table 2). Another step in defining the initial conditions was the problem of a loading operation of a suprastructure in accordance with the real conditions (Figure 2); therefore, according to the reference information, a maximum force of 100 N was used, and distribution pattern was considered as time-dependent (Figure 3). A gap (or distance) between the head screw and its seat was considered as screw loosening. The FEM basic design of implant models was determined using the CAD system. This system is able to display models three-dimensionally. CAD files are stored in the Catpart format that can be transferred to the FEM software. FEM data collection was carried out by ANSYS software. Interface meshing was carried out by a three-dimensional tetrahedral solid element, assuming that the implant was located in the lower left molar region. The X, the lower left molar, and Z axes were considered in the mesiodistal, longitudinal, and buccolingual directions, respectively. Nonlinear dynamic analysis was used by FEM to collect the implant system’s response. The axial pulse force of L1 was applied to the occlusal surface of the suprastructure. The movements associated with the abutment screw loosening were evaluated by displacements obtained from 4 points (mesial, distal, buccal, and lingual) at the abutment–implant interface.

**Table 1:** The steps for defining the interfaces between the implant components

**Model (A4, B4)> Connections>Contact Tool**	
**Name**	**Contact Side**
**Frictional-Implant to Screw**	**Booth**
**Frictional-Implant to Abutment**	**Booth**
**Frictional-Screw to Abutment**	**Booth**
**Bonded-abutment to Superstructure**	**Booth**

**Table 2:** Mechanical properties

**Materials**	**Young’s modulus ( GPa)**	**Poisson ratio**
**Titanium abutment and implant and screw**	**115**	**0.35**
**Ni-Cr Superstructure**	**150**	**0.26**

## Results

**Figure 4:**
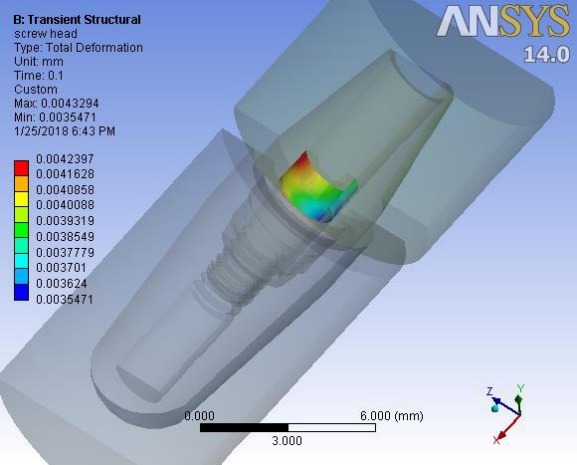
The octagon screw head displacement under vertical loading at 0.1 second.

**Figure 5:**
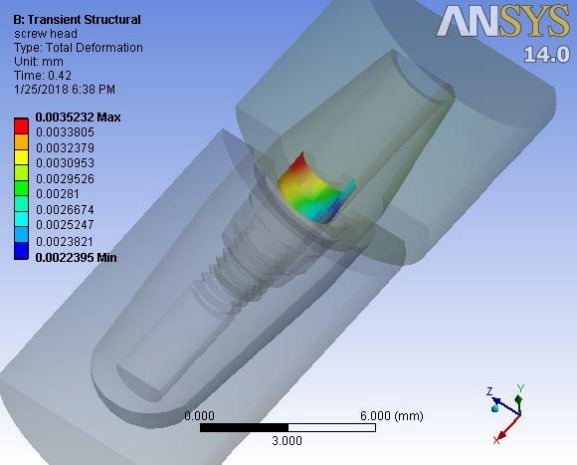
The octagon screw head displacement under vertical loading at 0.42 second.

**Figure 6:**
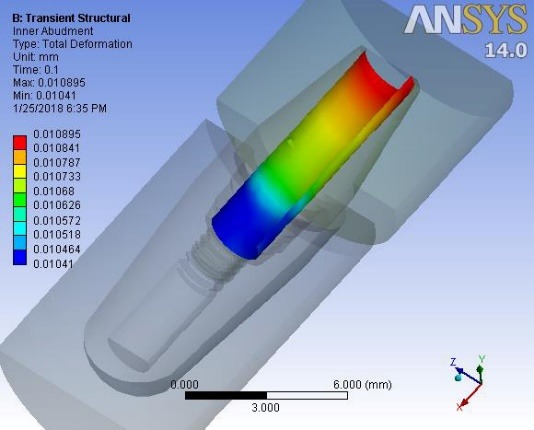
Displacement of the internal surface of the octagon abutment under vertical loading at 0.1 second.

**Figure 7:**
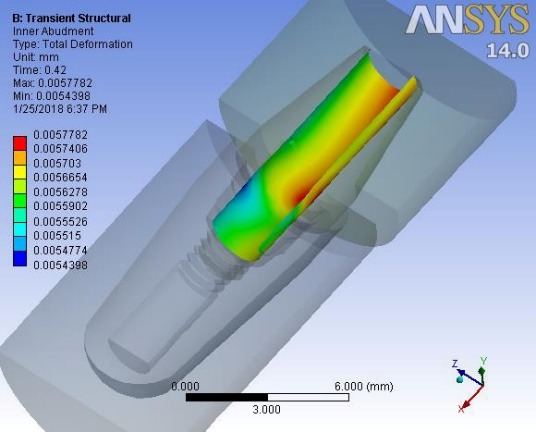
Displacement of the internal surface of the octagon abutment under vertical loading at 0.42 second.

**Figure 8:**
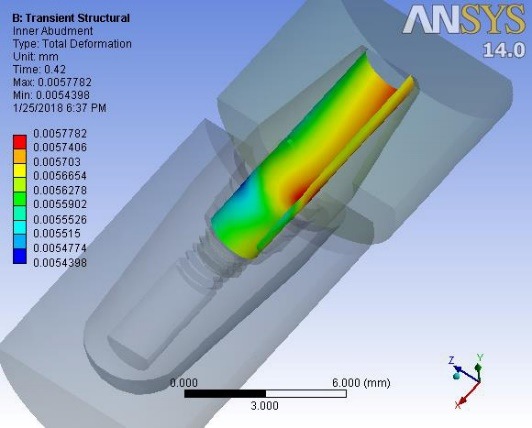
Displacement of the octagon connection interface under vertical loading at 0.1 second.

**Figure 9:**
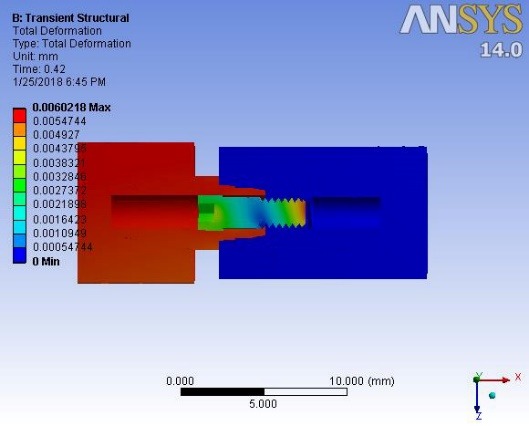
Displacement of the octagon connection interface under preload at 0.42 second.

Deformations were investigated at 0.1 second when a maximum force of 100 N was applied to the suprastructure and at 0.42 ssecond when no force was applied to the suprastructure. An average downward displacement of about 3.8 μm was obtained in an octagon connection at 0.1 second. This deformation is due to the application of a 400-N preload (3 N.cm torque) to the screw, as well as a 100-N force to the suprastructure (Figure 4). An average downward displacement of about 2.8 μm was recorded in the octagon head screw at 0.42 second. This deformation was due to a 400-N preload, which is equivalent to a 3-N.cm torque applied to the screw (Figure 5). An average displacement of about 10.7 μm was recorded in the internal surface of the abutment at 0.1 second, which was due to the application of a 100-N force on the suprastructure (Figure 6). An average displacement of about 5.6 μm was recorded in the internal surface of the octagonal abutment at 0.42 second, which was due to the application of a preload of 100 N on the screw (Figure 7). The displacement of all the components of the octagon connection at 0.1 second was about 7 μm greater than the screw head displacement, indicating that the head screw was detached from the abutment surface under this loading (Figure 8). The displacement of all the components of the octagon connection at 0.42 second was approximately 6 μm, and the screw head displacement was about 5 μm. The screw head caused the displacement in the abutment and suprastructure under the preloading conditions and pulled it along about 6 μ toward the bone (Figure 9).

**Figure 10:**
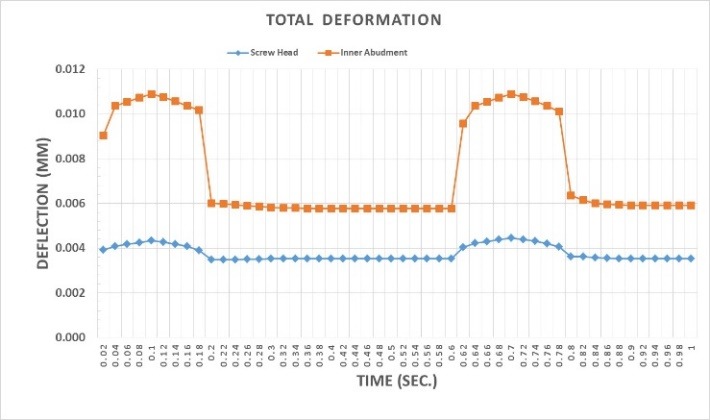
Comparison of the maximum displacement of the screw head and the internal part of the octagon abutment at their interface during preloading and vertical loading.

**Figure 11:**
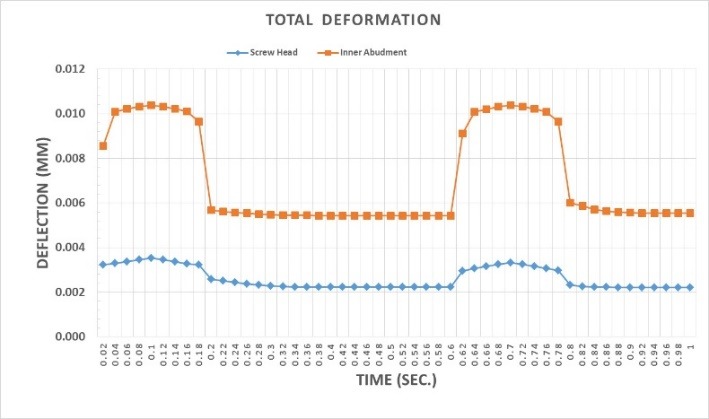
Comparison of minimum displacement of the screw head and the internal part of the octagon abutment at their interface during preloading and vertical loading.

When loading was carried out on the suprastructure from 0 to 0.1 second and from 0.6 to 0.8 second, the displacement of the octagonal abutment increased as compared to the screw head, resulting in the separation of the screw surface from the internal surface of the abutment (Figure 10). At other times, the preload and torque applied to the screw led to the displacement of the screw head, and eventually, the displacement of the abutment–suprastructure interface directed them toward the jawbone (Figure 11). The mean downward displacement value of the screw head of the morse hex connection at 0.1 second was about 8 μm. This deformation resulted from a preload of 400 N (equivalent to a 3-N.cm torque) applied in the screw, as well as a force 100 N applied to the suprastructure. The mean internal surface displacement of the abutment of the morse hex connection, which was connected to the screw head at 0.1 second was about 8.4 μm, due to the application of a 100-N force on the suprastructure. The mean screw head displacement of the morse hex connection at 0.42 second was 7.6 μm downward, which was due to the application of a 30-N.cm torque. The mean internal surface displacement of the abutment of the morse hex connection that was in contact with the screw head at 0.42 second was about 5.7 μm, due to the application of the preload force on the screw. Displacement of all the components of the morse hex together at 0.1 second and screw head displacement was about 9 μm, indicating that under this loading, the head screw was not separated from the abutment surface. Displacement of all the components of the morse hex together at 0.42 second and screw head displacement was about 6 and 7 μm, respectively. The screw head caused the above displacements in the abutment and suprastructure when the preload was used and extended it along the bone by about 7 μm. When loading was being applied on the suprastructure at 0 to 0.1 second, as well as 0.6 to 0.8 second, the morse hex abutment displacement was roughly similar to that of the screw head with a 1-μm discrepancy. Therefore, the relative separation of the screw surface from the internal surface of the abutment did not occur. The torque applied to the screw at other times led to the displacement of the screw head, ultimately displacing the abutment–suprastructure interface toward the jawbone, and the displacement rate was still the same for both parts.

**Figure 12:**
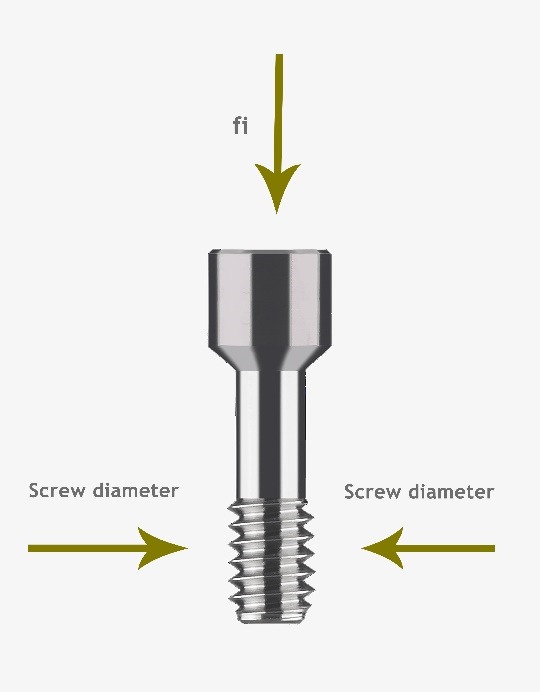
Illustration of force applied to the screw.

## Discussion

The present study investigated the rate of screw loosening in two different types of implant connection, using the finite element method (FEM). The results showed different rates of screw loosening in the two types of connection during the application of 30-N.cm torque in the different types of connection used. The types of the implant–abutment connections were also effective in the screw loosening rate. Complications of an implant-supported prosthesis can be divided into two categories of mechanical and biological problems, a significant portion of which is attributed to screw loosening.^11^ The present study aimed to determine the effect of the type of implant–abutment connection and its geometry on screw loosening. External forces always lead to transient dynamic deformations in the screw connection. The misfits or deformations already present in the implant components increase the rate of screw loosening. The weak interface between the implant components increases the initial displacement and causes abrasion in the connecting areas, which also increases the distance in the screw connection region. The application of force might lead to the creation of tensile forces in the screw and contribute to screw loosening.^11^ Only two-piece abutments and cemented restorations were used in the present study. According to the Bickford study, failure in the screw connection region occurs in two stages. In the first stage, the external functional forces gradually reduce the clamping force, and then the remaining clamping force is rapidly reduced.^11^ In the present study, a different degree of abutment screw loosening occurred inside the implant at 30-N.cm torque in the selected connections, with the lowest rate observed in the morse hex connection. Two types of connecting methods, including screw and interface with fit taper, which is also called morse taper, are commonly used to connect an abutment to an implant. In screw-based systems, the implant–abutment connection depends on the screw preload. However, morse taper interfaces depend on long contact pressure and frictional resistance. In screw-dependent connections, mechanical complications, such as screw loosening or creep at the screw–implant interface, can lead to clinical complications. However, in cases where morse taper connections are used, the abutment loosening is less problematic.^12^ Limited data is available on the number of cycles needed to loosen the screw, especially in the oral cavity, and the presence of biological tissues, such as bone, periodontal ligaments, and temporomandibular joint with differences in modulus of elasticity, has made the analysis more complicated. In addition, the screw-related factors, such as the screw yield point, the type of screw used, the duration of the use of the screw, and its potential for fatigue, affect screw loosening. Therefore, the potential of screw loosening is variable, and a large number of related factors are still unknown.^13^ The screw material can also be effective in the amount of the resultant preload. The tensile strength and yield point for the gold-redesigned screws are greater than the former titanium screws. Therefore, a higher preload can be made in gold alloy screws.^8^ Therefore, in this study, we used one screw type in both types of connection to create similar conditions. Static and dynamic forces are used to analyze micro-gap formation at an implant–abutment interface. Zipprich et al^14^ analyzed ten different systems of implant and concluded that connections with a clearance fit showed micro-motion under a static force of 200 N, although the same force did not create any micro-motion in the precise conical connections, such as Astra Tech and Ankylos.^14^ Micro-motion is an important parameter that has received relatively little attention in the implant-supported prosthetic field. It is necessary to consider this parameter as a long-term success factor for implants, which can predict the initial stability of implants within the bone and the stability of the implant components. Occlusal forces, which occur during actions such as clenching and chewing, are also transmitted to the components of the dental implants, leading to movements between the implants and abutments.^14^ The present study investigated three-dimensional models to evaluate the micro-motion of implant components under simulated occlusal forces. Overall, different connection forms showed different patterns of micro-motion. The results of the present study revealed the highest and lowest micro-motions in the morse hex and octagon connections, respectively. The micro-motion distribution pattern in abutments with morse hex connection was different from that of other connections, attributable to the geometry of this connection, which is of polygonal and cylindrical shapes in the coronal and apical regions, respectively.^14^ Ameen Khraisat et al assumed that the twist resulting from the lateral exterior central loading could be countered by the clamping force created at screw–hexagon rotational interface, although it was shown that a 32-N.cm clamping torque cannot completely counteract the external torque forces. The size of the rotation created by the external central forces might depend on the rotational abutment freedom, considering the role of the hexagon and the frictional forces produced between the surfaces by the clamping forces. The interaction of these forces produces a reversible hexagonal internal rotation in the abutment against the ​​corresponding region involved in the implant. A larger lateral micro-movement in the abutment leads to a greater loss of the preload. An important result of the rotational misfit in these single implant-supported restorations is the susceptibility to these micro-movements. A better fit between the implant components will result in greater stability of the implant.^8^ According to the results of the present study, the lowest rate was observed in the morse-hex connection, one of the possible reasons for which could be a higher fit in the morse-hex connection than the other connection, which results in lower preload loss. The relationship between the amount of torque (torsional moment) and the approximate values of the resulting preload force are calculated for the usual screws (with a friction coefficient of 0.05). The 0.2 coefficient might vary depending on the amount of torque and friction.

### The preload force from T-screw cement

(1) $$T=0.2F_{i}d\;or\;F_{i}=\frac{T}{d}\times 5$$

The momentum applied in the assumption of the problem is considered to be

(2) $$T=30N.cm\Rightarrow F=\frac{30N.cm}{0.2cm}\times 5=750N$$

According to the approximate formula, a 750-N preload is obtained using the above approximate formula.^15^ However, according to available resources, the amount of preload force is considered to be about 400 N, which was also used in the present study to solve this problem according to the formula proposed in the articles. The rate of screw deformation caused under the preload conditions is calculated as follows (A fully documented mechanical equation). This equation is fully correct when there is no material under the screw head.^11^

(3) $$\delta =\frac{F_{i}L}{E.A}$$

Fi: Preload force

L: Screw length

E: Screw modulus of elasticity (Young)

A: Cross-section

The deformations are calculated for these two types of implants.

(4) $$\begin{array}{l} \delta_{1}=\frac{400\times 5\times 10^{-5}(m)}{3.14\times 10^{-6}(m^{2})\times 115\times 10^{4}N/m^{2}}=5.5\mu m\\ L_{2}=5mm,\delta_{2}=7.5\mu m,L_{2}=6.5mm,\\ \delta_{3}=6.5\mu m,L_{3}=7.77mm, \end{array}$$

The morse hex taper connection^2^ was considered in the equations used to calculate the octagon connection.^1^ Since the screw of the two samples was different due to the internal conical geometry of the abutment, and different rates of screw deformation were thus recorded in the two above-mentioned samples, it should be noted that the same results were also observed in FEM analyses. However, since different materials were used under the screw head in different samples, the values would not be obtained in accordance with the above equation. However, the results obtained by the ANSYS analysis were close to the aforementioned calculated values. Considering the exact and somewhat realistic modeling carried out in ANSYS, the resultant values are more accurate, and the above calculations are only given to show approximate estimates.

Practically, considering the complex geometry of the implant components, it is even impossible to calculate the rate of the screw deformation caused by preload and other components affected by its use of very similar complex formulas. Therefore, analytical softwares, such as ANSYS, are used. However, what is perceived in practice to understand the cause of the implant components’ static deformations is obtained from the general formula below.^15^

(5) $$\begin{array}{l} F_{i}=\delta (\frac{EA}{L})=\delta .k\;or\;\delta =F_{i}(\frac{L}{EA})\\ F_{i}=\delta .k \end{array}$$

The value in the parentheses shows the stiffness or springiness amount. The preload value is the same in all the three implant samples that were studied (F_i_ = 400 N). So why is the rate of deformations (δ1 = 2δ) not the same? Certainly, according to the formula, this is attributed to the difference in the interface stiffness. Since the material of both interfaces is identical (E_1_ = E_2_), the conic inside of the abutment is not of the same screw length, and the length of the implant components is the same (L_1_= L_2_); therefore, the remaining difference is related to the cross-section of the implants in the area on which the force was applied. In fact, the different geometries of the implants have created different cross-sections under the screw and created different stiffness levels. As the cross-section increases (A↑), the stiffness level ($(E\frac{A}{L})k↑$) has less deformation in the constant preload(.

$$F_{i}=cte=k↑.\delta ↑F_{i}=cte$$

However, in the case of screws, the change in the length of the screws causes different stiffness levels and different deformations. The same result was exactly observed in the analysis L↑→k↓→δ↑; however, due to the complex surface of each implant, only ANSYS software is used to calculate the exact rate of deformations.

Finally, two important deformations were observed in each implant interface: one for the screw head and one for the abutment interface. Therefore, it can be concluded that the separation of the screw head from the internal surface of the abutment depends on the relative deformation between the screw head and the internal surface of the abutment. As explained, the screw deformation depends on its length with the same cross-section and material, and the abutment deformation depends on its cross-section with the same length and the same material.

In the morse hex taper interface, the longer screw length causes more deformation in the screw head. In addition, the abutment deformation due to its cross-section and specific geometry resulted in a deformation in which the screw head did not separate from the internal surface of the implant; however, this has somewhat happened in the case of the octagon. It can be concluded that the lower the screw springiness (the longer it is), the lower the abutment deformation (for example, the higher cross-section); and the higher its springiness, the lower the degree of separation and screw loosening.

## Authors’ contributions

The study was planned by AP and SJA. The literature review was performed by RN, and VG contributed to writing the manuscript and English editing. The statistical analyses and interpretation of data were carried out by RN. VG contributed to the publication of the article and developing the protocol. SJ and AT contributed to the development of the protocol. SJA contributed to the thesis, article writing, and publication. All authors have read and approved the final manuscript.

## Acknowledgments

Hereby, Dr. Mahboobkhah is appreciated for his sincere guidance.

## Funding

This study was supported by the Vice Chancellor for Research (VCR), Faculty of Dentistry, Tabriz University of Medical Sciences (TUOMS), Tabriz, Iran.

## Conflict of interests

The authors declare no conflict of interests with regards to the authorship and/or publication of this article.

## Ethics approval

Not applicable.
